# ADHD symptoms and use of anabolic androgenic steroids among male weightlifters

**DOI:** 10.1038/s41598-022-12977-w

**Published:** 2022-06-08

**Authors:** Emilie Kildal, Bjørnar Hassel, Astrid Bjørnebekk

**Affiliations:** 1 Department of Neurohabilitation, Oslo University Hospital, Kirkeveien 166, 0450 Oslo, Norway; 2 Institute of Clinical Medicine, University of Oslo, Oslo, Norway; 3 The Anabolic Androgenic Steroid Research Group, Section for Clinical Addiction Research, Oslo University Hospital, Oslo, Norway

**Keywords:** Neuroscience, Psychology, Endocrinology, Neurology, Risk factors

## Abstract

Use of anabolic androgenic steroids (AAS) is associated with adverse health effects. The factors that predispose to AAS use among athletes are poorly understood, but attention deficit/hyperactivity disorder (ADHD), which is known to occur among athletes more often than in the general population, is associated with risk behaviors, including substance abuse. We aimed to see if AAS use in male weightlifters was associated with ADHD symptoms, and test the link between ADHD symptoms and cognitive performance. Hundred and forty male weightlifters, 72 AAS users and 68 weightlifting controls (WLC), completed the Achenbach system of empirically based assessment (ASEBA) for ADHD symptoms and underwent cognitive examination. Self-reported ADHD symptom scores were significantly higher among AAS users compared to WLC, and scores in the range indicating clinically important ADHD was significantly more common in the AAS-using group. Age of onset of AAS use correlated inversely with ADHD scale score (r = − 0.35; p = 0.003). ADHD score correlated inversely with cognitive scores for working memory (r = − 0.25, p < 0.001), processing speed (r = − 0.24, p < 0.001), verbal learning and memory (r = − 0.19, p = 0.03), and problem solving (r = − 0.20, p = 0.02). AAS use among weightlifters is associated with ADHD symptoms and corresponding lower cognitive performance. Recognising a relationship between ADHD symptoms and AAS use may guide drug prevention strategies in sports.

## Introduction

Use of anabolic androgenic steroids (AAS) is a serious abuse problem among professional and recreational athletes^1–4^. AAS have anabolic properties, stimulating muscle growth^5^, and androgenic properties inducing masculine secondary sexual characterisics, and augments cognitive features like alertness^6–9^. However, AAS use may have serious psychological and physiological consequences, such as major mood syndromes and cardiovascular disease^10,11^. The main activity of AAS in the brain occurs via activation of widely distributed cytoplasmic androgen receptors, as has been shown in animal studies^12–15^. This may explain the various effects that AAS have on cognition and mental state^10,11,16,17^. Long term AAS use is associated with both structural brain abnormalities^18–21^ and cognitive and behavioral abnormalities^20,22,23^. Several studies suggest an association between AAS use and aggressiveness, hostility, mood swings, and violent crime^3,18,24–31^. Still, its massive impact on muscle growth has made AAS popular among athletes worldwide^32–34^.

The impact of AAS doses may be difficult to determine for several reasons. More than 100 different AAS compounds have been synthesised, with three major classes that differ in molecular structure and metabolic half-lives, and hence physiologic effects. AAS include testosterone and its various synthetic derivatives with the three most common forms being (1) 19-nortestosterone derivatives (nandrolone phenylpropriate, nandrolone decanote, methenolone enanthate), (2) C-17 β-ester derivatives (testosterone propionate, cypionate, enanthate, or undecanoate), and (3) 17 α-alkyl derivatives (stanozolol, oxymetholone, norethandrolone, danazol). Weightlifters commonly coadminister various AAS and administer drugs in cycles of use and nonuse lasting from weeks to months^1,22,35–37^.

The factors that predispose to AAS use are poorly understood. However, attention deficit/hyperactivity disorder (ADHD) occurs among athletes at different levels, from any organized sport to the elite, with a prevalence between 7 and 11%, higher than in the general population^38–40^. Moreover, in a longitudinal study of 100 AAS users, 17% reported a history of psychiatric illness at inclusion, where ADHD was the most common diagnosis reported by 7%^30^. Persons with ADHD have increased risk of substance use^41–46^, which, theoretically, could include AAS use. ADHD implies inattention, and/or impulsivity and hyperactivity at a disabling level. Symptoms at levels that do not meet the diagnostic criteria for ADHD may still affect a person’s cognition and behavior. The severity and number of ADHD symptoms are associated with the degree of psychiatric comorbidity and disability, including cognitive abnormalities^47^. Cognitive domains commonly affected in ADHD include attention, working memory and problem solving^48–50^. As mental health issues may be neglected among athletes^51^, adverse symptoms, like neurocognitive deficits, may be present despite the lack of diagnosis and treatment. While the sporting context might serve as an outlet for certain symptoms, these athletes may suffer significantly in other contexts like in social relationships or working life. As ADHD is a risk factor for overall drug use^41–46^, and ADHD symptoms are common yet often undetected among athletes^38–40^, it is possible that ADHD symptoms could predispose to AAS use.

The aim of our study was to see whether use of AAS among male weightlifters is associated with symptoms of ADHD. To identify ADHD symptoms participants were asked to complete the Achenbach System of Empirically Based Assessment (ASEBA) questionnaire. We further examined how self-reported ADHD symptoms were associated with cognitive performance as evaluated by cognitive examination.

## Methods

### Study population

The study was conducted at the Department of Physical Medicine and Rehabilitation, Oslo University Hospital, Oslo. Two groups of weightlifters over 18 years of age were recruited to the study: those with (1) current or previous use of AAS, with at least 1 year of cumulative AAS exposure (n = 89), or (2) no previous or current exposure to AAS or other muscle- and performance-enhancing drugs (n = 72). Cognitive data was obtained from 159 participants, including 89 current or previous AAS users, and 70 weightlifting controls (WLC). Of those, 24 were not included in the current study due to missing data on the ASEBA questionnaire, and one was excluded due to a neuroradiological finding and one due to epilepsy. The present study comprises 134 male weightlifters; thereof 72 AAS users and 62 WLC with complete datasets including cognitive tests and the ASEBA questionnaire for ADHD symptoms. The sample is partly overlapping with the one described in our previous work^19,22^.

The recruitment was done through websites or online forums associated with heavy resistance training or AAS use, as well as through posters and flyers distributed in selected gyms in Oslo. Every participants received a written description of the study prior to participation and written formal consent was obtained from all participants. They were compensated with 1000 NOK (approx. 125 USD) for their participation. The study was approved by the Regional Committees for Medical and Health Research Ethics South East of Norway (approval # 2013/601), and all research was carried out in accordance with the Declaration of Helsinki.

### Cognitive assessment

Participants underwent eight neuropsyhological tests covering a broad range of cognitive domains, including the Wechsler Abbreviated Scale of Intelligence^52^, the California Verbal Learning Test^53^, Letter Memory Task^54,55^, the Delis-Kaplan Executive Functioning’s Color-Word Interference Test (CWIT) and Trail Making Test (TMT) from the Delis-Kaplan Executive Functioning (D-KEFS) test battery^56^, and Corsi Block Test from the PEBL (Psychology Experiment Building Language) Version 0.13 test battery^57,58^. The twenty-five subtests were divided into six cognitive domains with acceptable reliability as described by Bjørnebekk et al.^22^. Of those, five were considered relevant for the current study; (1) Speed, (2) Working memory, (3) Learning and memory, (4) Problem Solving, and (5) Executive functioning. Overview of the cognitive tests administered, and the cognitive domains are shown in Table 1.

**Table 1:** Tests used for cognitive assessment and what functions they assess.

Test name	Abbreviation	Measures included	Function measured
California learning test-second edition	CVLT-II	Total learning, delayed recall 30 min, and false positives	Learning and memory
Wechsler abbreviated scale of intelligence	WASI	Matrix reasoning	Problem solving
Stroop color-word interference test—Delis Kaplan	CWIT—D-KEFS	CWIT1-4 (color naming speed, word reading speed, inhibiton, switching), contrast and flexibility	Executive function, Processing speed
Trail making test—Delis Kaplan executive function	TMT—D-KEFS	TMT1-4 (visual scanning, number sequencing, letter sequencing, number-letter switching)	Executive function, Processing speed
Letter memory task	Letter memory	Letter memory	Working memory
Corsi block—tapping task—psychology experiment building	Corsi, PEBL	Block span and memory span	Working memory
Attention network task	ANT	Response time	Processing speed

### Assessment of ADHD symptoms

The Adult Self Report (ASR) ASEBA is a revision of the Young Adult Self-Report protocol for adults aged 18–59 originally derived from the widely used Child Behavior Checklist^59^. The ASEBA assesses emotional and behavioral problems in a standardised format and has performed well in validation studies (sensitivity = 68.7% and specificity = 99.5%) with high concordance with clinician diagnosis^42,59–63^. The ASR ASEBA contains 126 items to assess behavior that have occurred over the past 6 months with the total score for each scale being the sum of the scores for scale items. On all scales a T score > 65 is clinicaly concerning, while a T score > 70 is indicative of diagnosis^59^.

### Data presentation and statistics

Data are given as mean and standard deviation (SD) values or as number of participants and percentage, as appropriate. Statistical analyses were performed using SPSS version 25^64^ and violin plot using R ggplot2^65^ Group differences in demographic data and ADHD scores were evaluated with two-tailed independent sample *t* tests ot assuming equal variance and Fisher’s exact tests for categorical data. Differences in ADHD scores were evaluated with Wilcoxon–Mann–Whitney tests, to account for the non-normal distributions. To explore whether ADHD symptoms seems to be influenced by current use of AAS, a similar analysis within the AAS-group was conducted comparing current and past AAS users (defined as more than 1 year since past AAS use). Lastly, Spearman’s rank-order correlation (*r*_s_) was used to investigate relationships between ADHD symptoms, cognitive performance, and parameters of AAS use. For cognitive performance z-transformed residuals were used, removing variability associated with age and education.

## Results

The group of AAS users (n = 72) and WLC (n = 62) weightlifters did not differ significantly with respect to age or height (Table 2). The two groups did however differ on weight, time spent excercising, and bench press record where AAS users were significantly heavier, and had higher bench press records compared to the WLC even though they spent less time per week on strength training. They also differed on average IQ score and years of education: WLC had significantly longer education and higher IQ scores.

**Table 2:** Demographic and clinical data.

Sample characteristics	AAS (n = 72)		WLC (n = 62)		t	p
	Mean	SD	Mean	SD		
Age, years	33.2	8.2	31.0	9.3	− 1.34	0.091
Education, years	14.3	2.5	15.8	2.7	3.26	< 0.001
IQ	105.6	11.8	113.3	9.5	4.15	< 0.001
Alcohol, units/week	1.6	3.2	3.4	5.0	2.34	0.020
Height, cm	180.9	6.6	181.0	6.9	0.09	0.842
Weight, kg	97.6	13.7	90.9	14.6	− 2.72	0.007
BMI	29.7	4.1	27.7	4.1	− 2.8	0.6
Cigarettes per day	1.5	1.5	0.3	2.6	− 1.95	0.051
Strength training, min/week	346.1	184.7	479.7	246.8	3.55	0.037
Bench press record, kg	169	31	135.4	33	− 5.88	< 0.001

Male weightlifters who used AAS (n = 72) were compared to a group of weightlifters who had not used AAS (n = 62). Data are number of participants and mean values (SD) for all variables.

AAS: anabolic androgenic steroid, WLC: weightlifting control subjects, IQ: intelligence quotient, BMI: body mass index.

The two groups differed on substance use other than AAS. Alcohol consumption was lower among AAS users, while tobacco use was more frequent among AAS users (Table 2). Use of anxiolytics and antidepressants were more common among AAS users, as 32% (n = 23) reported to have used these medications compared to only 3% (n = 2) of WLC (p < 0.001).

Participants’ AAS use typically started in their early twenties (mean age 21.5 years, SD = 5.3, range 12–39) and on average AAS had been used for 9.5 years (SD = 5.6, range = 1.5–30).

**Figure 1:**
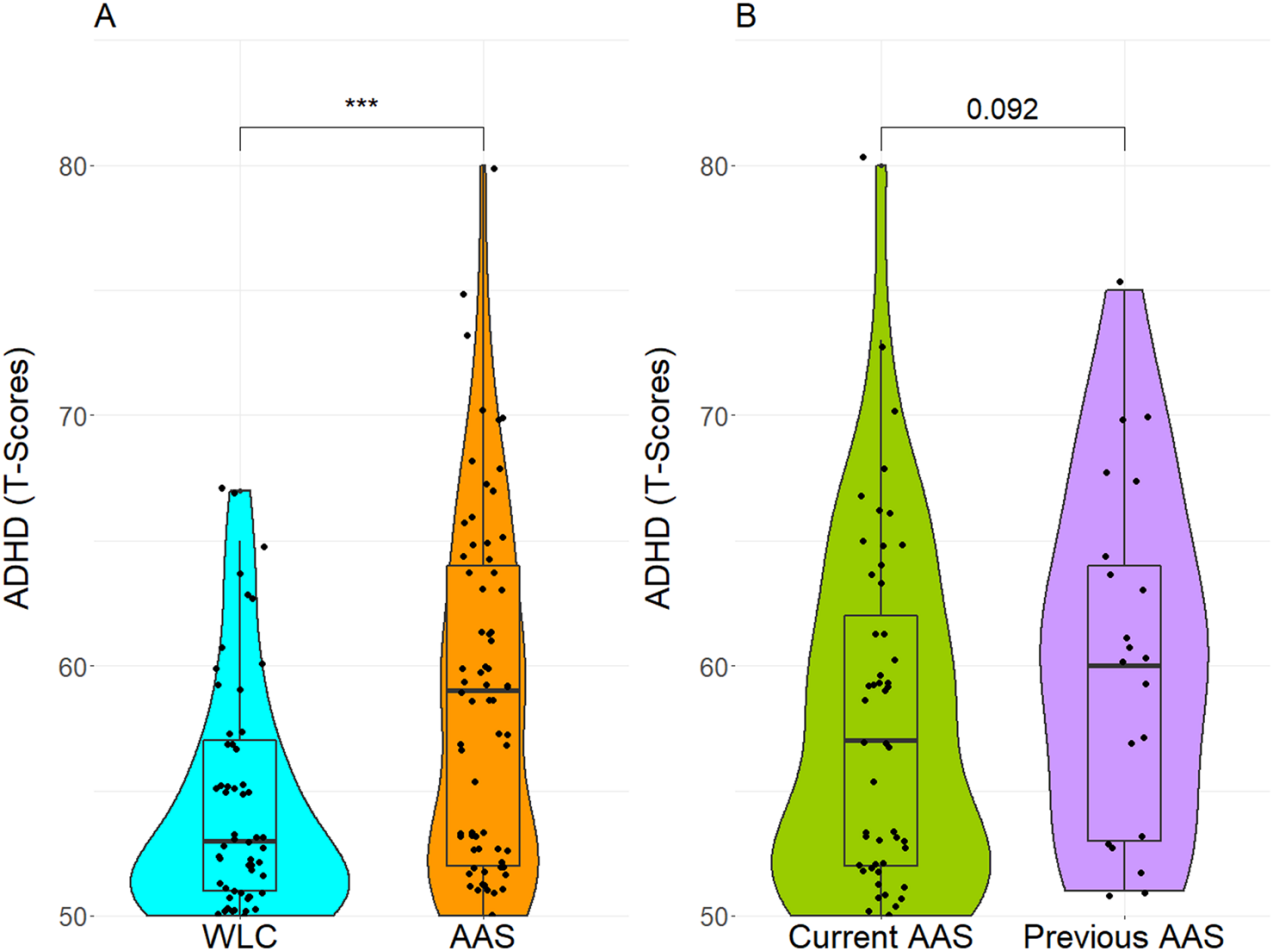
ADHD symptoms among AAS users and WLC. AAS-using male weightlifters (n = 72) and WLC (n = 62) complete the ASEBA questionnaire on ADHD symptoms. (**A**) Shows ADHD symtoms presented as T scores in AAS users and controls. (**B**) Shows ADHD symptoms presented as T scores in current and previous users of AAS. ADHD: attention deficit/hyperactivity disorder, WLC: weightlifting controls, AAS: anabolic androgenic steroids.

ADHD symptom scores were higher among AAS users (*Mdn* = 59.0) than among WLC (*Mdn* = 53.0). This difference was statistically significant, *U (N*_AAS_ = 72, *N*_*WLC*_ = 61,) = 1360.00, *z* = − 3.79, *p* < 0.001 (Fig. 1A). Also, there was a significant group difference in the frequency of clinically concerning ADHD symptoms (t-score > 65), where twelve (16.7%) AAS-users had scores within the borderline or clinical range, compared to two (3.3%) of the WLC (p = 0.02). A negative correlation was found between age of onset of AAS use and self-reported ADHD symptoms (*r*_*s*_ = − 0.35, *p* = 0.003), whereas years of AAS use were not related to ADHD scores (*r*_*s*_ = 0.08, p = 0.50). Furthermore, analyses within the AAS sample, showed that ADHD scores of current users (*Mdn* = 57.0) were lower than scores of previous users (*Mdn* = 60.0), however not significantly, *U (N*_*AAS CURRENT*_ = 51, *N*_AAS PAST_ = 21,) = 399.50, *z* = − 1.69, *p* = 0.09 (Fig. 1B).

Self-reported ADHD symptoms correlated inversely with cognitive scores on working memory, processing speed, verbal learning and memory, and problem solving (Table 3). In contrast, no correlation was found between self-reported ADHD symptoms and executive function.

**Table 3:** Spearman’s correlation between self-reported ADHD symptoms and neuropsychological test scores.

**ADHD**	Working memory	Speed	Verbal learning and memory	Problem solving	Executive function
Rho	− 0.25	− 0.24	− 0.19	− 0.20	0.11
P	0.00	0.00	0.03	0.02	0.21

The participants neuropsychological test scores within five cognitive domains were correlated to their ADHD T scores. Correlation is corrected for age and education using standardised residuals. The data are Spearman’s correlation coefficients (Rho) and their corresponding p values.

## Discussion

The main finding of the present study was the higher occurence of ADHD symptoms among AAS-using male weightlifters compared to WLC. This prevalence is likely an underestimation (due to ASEBA’s low sensitivity (68.7%) for ADHD symptoms^66^. Other studies have found that ADHD entails a risk for substance abuse^46,67–70^. Our finding suggests that this risk also includes use of AAS. As the psychological and physiological effects of AAS use include adverse effects like major mood syndromes, hostility, structural and functional brain abnormalities^19,21,22^, violent crime, and cardiovascular diseases^10,11^, prevention programs are needed. Treatment and medication for ADHD have been shown to prevent substance abuse^44,71,72^. In-person brief motivational interventions, programs with discussion of sports nutrition, exercise alternatives to AAS, drug refusal role-playing, and the creation of health promotion messages have been shown effective in drug prevention among athletes^73,74^. Recognising the relationship between ADHD symptoms and AAS use can inform such prevention programs in sports medicine.

We found that ADHD symptoms correlated inversely with age of onset of AAS use. This cross-sectional study is not able to determine whether the ADHD symptoms were the cause or the consequence of AAS use, or whether AAS use caused ADHD symptoms. On the one hand, ADHD is present from childhood^75^, whereas AAS-exposure occurs later in life, an observation suggesting that ADHD may be the primary factor for AAS use. On the other hand, AAS at high doses are known to cause impulsivity and aggressiveness^9^, two symptoms that are common in ADHD^76^. In the present study, three observations suggested a primary role for ADHD as predisposing to AAS use. First, the degree of self-reported ADHD symptoms did not increase with the number of years of AAS use, suggesting that greater length of AAS use does not increase ADHD symptom score. Second, the age of onset of AAS use was inversely correlated with ADHD symptom level, suggesting that the more severe the ADHD symptomatology, the greater the likelihood of early AAS onset. Third, previous users of AAS scored equally high as current users, suggesting that current AAS use does not increase the severity of ADHD-like symptoms. This conclusion fits the notion that ADHD predisposes to substance abuse in general^41–46^. However, prospective studies are needed to determine to what degree ADHD predisposes to AAS use and whether AAS use may cause the appearance of ADHD symptoms.

We found a higher use of antidepressants and anxiolytics among AAS-using male weightlifters than among WLC with respect to. This finding is in accordance with previous studies reporting high rates of psychiatric comorbidity in ADHD^77–83^. It should be noted, however, that the majority of AAS users did not use prescription drugs, whether for physical og psychological conditions. Thus, the use of psychotropic medication among the AAS users was not a major confounder in our study.

ADHD scores correlated inversely with scores on several tests of cognitive domains related to ADHD. This finding indicates that the self-reported symptoms of ADHD were reliable and that the ADHD symptoms had implications for the participants’ cognitive function. Specifically, we found that self-reported ADHD symptoms correlated inversely with cognitive scores on working memory, processing speed, verbal learning and memory, and problem solving. These findings are consistent with previous findings on cognitive deficits among persons with ADHD^41,84–88^. Executive functioning was the only cognitive domain measured that did not correlate with ADHD symptoms. Deficits in executive function is considered a central underlying mechanism of ADHD^89,90^. However, our findings are in accordance with studies of other patients and samples of AAS users, in which performance-based executive functions and self-reported measures of executive functions in everyday life are unrelated^23,91,92^.

Some limitations should be noted. First, the cross-sectional study design implies that we cannot draw definite conclusions about whether ADHD causes AAS use or vice versa. Because we limited ourselves to the study of male weight lifters, our findings are not generalizable to female AAS users. Further, as we have recruited participants from online forums, social media and gyms, targeting heavy resistance training and AAS use, we risk having a skewed selection of AAS users. Therefore, we cannot generalize from our study to subpopulations such as prisoners^93^, substance use patients^94^, and sexual minority males^95^, among whom AAS use also occurs. It is also possible that our offering financial compensation for participation could introduce recruitment bias. However, the modest sum of money that participants received was intended to compensate for their use of time and their travel expenses when going to the hospital. Finally, whereas we did ask about the use of medications, we did not ask about ADHD medication specifically. Therefore, we do not know to what degree use of ADHD medication influenced our results.

## Conclusion

Our findings suggest that ADHD symptoms are more common among weightlifters who use AAS. Correspondence between ADHD symptoms and cognitive test performance substantiated this finding. Recognising a relationship between ADHD symptoms and AAS use may guide prevention strategies against AAS use in sports.

## Abbreviations

AAS: Anabolic androgenic steroids
WLC: Weightlifting controls
ADHD: Attention deficit/hyperactivity disorder
ASEBA: Achenbach system of empirically based assessment
